# Epitope Flexibility and Dynamic Footprint Revealed by Molecular Dynamics of a pMHC-TCR Complex

**DOI:** 10.1371/journal.pcbi.1002404

**Published:** 2012-03-08

**Authors:** Cyril F. Reboul, Grischa R. Meyer, Benjamin T. Porebski, Natalie A. Borg, Ashley M. Buckle

**Affiliations:** 1 Department of Biochemistry and Molecular Biology, Monash University, Victoria, Australia; 2 ARC Centre of Excellence in Structural and Functional Microbial Genomics, Monash University, Victoria, Australia; 3 Monash eResearch Centre, Monash University, Victoria, Australia; Utrecht University, Netherlands

## Abstract

The crystal structures of unliganded and liganded pMHC molecules provide a structural basis for TCR recognition yet they represent ‘snapshots’ and offer limited insight into dynamics that may be important for interaction and T cell activation. MHC molecules HLA-B*3501 and HLA-B*3508 both bind a 13 mer viral peptide (LPEP) yet only HLA-B*3508-LPEP induces a CTL response characterised by the dominant TCR clonetype SB27. HLA-B*3508-LPEP forms a tight and long-lived complex with SB27, but the relatively weak interaction between HLA-B*3501-LPEP and SB27 fails to trigger an immune response. HLA-B*3501 and HLA-B*3508 differ by only one amino acid (L/R156) located on α2-helix, but this does not alter the MHC or peptide structure nor does this polymorphic residue interact with the peptide or SB27. In the absence of a structural rationalisation for the differences in TCR engagement we performed a molecular dynamics study of both pMHC complexes and HLA-B*3508-LPEP in complex with SB27. This reveals that the high flexibility of the peptide in HLA-B*3501 compared to HLA-B*3508, which was not apparent in the crystal structure alone, may have an under-appreciated role in SB27 recognition. The TCR pivots atop peptide residues 6–9 and makes transient MHC contacts that extend those observed in the crystal structure. Thus MD offers an insight into ‘scanning’ mechanism of SB27 that extends the role of the germline encoded CDR2α and CDR2β loops. Our data are consistent with the vast body of experimental observations for the pMHC-LPEP-SB27 interaction and provide additional insights not accessible using crystallography.

## Author Summary

When pathogens replicate within a host cell, their proteins are degraded into peptides, which are captured by the major histocompatibility complex (MHC) and brought to the cell surface. The peptide-MHC (pMHC) is surveyed by T cell receptors (TCRs) expressed on the surface of T cells. If the peptide is foreign, the peptide-MHC-TCR interaction initiates an immune response to eliminate the pathogen. However, the combinations of pMHC and TCRs are diverse. We ask how TCRs discriminate between structurally similar pMHCs? We address this by focusing on two MHC molecules that differ by a single change, both bind the same peptide but only one instigates a dominant immune response. Intriguingly, the single difference between the two MHCs does not alter the peptide shape nor does it contact the peptide or TCR. We examined the flexibility of the pMHC-TCR interface using molecular dynamics simulations. We observed differences in the peptide and TCR flexibilities that could explain their contrasting physiologies, as well as clues to how the TCR moves atop the MHC in order to ‘scan’ it. Our analysis provides insight into a particular pMHC-TCR interaction not accessible using crystallographic methods, and indicate dynamics may play an influential and perhaps under-appreciated role in other pMHC-TCR systems.

## Introduction

**Figure 1:**
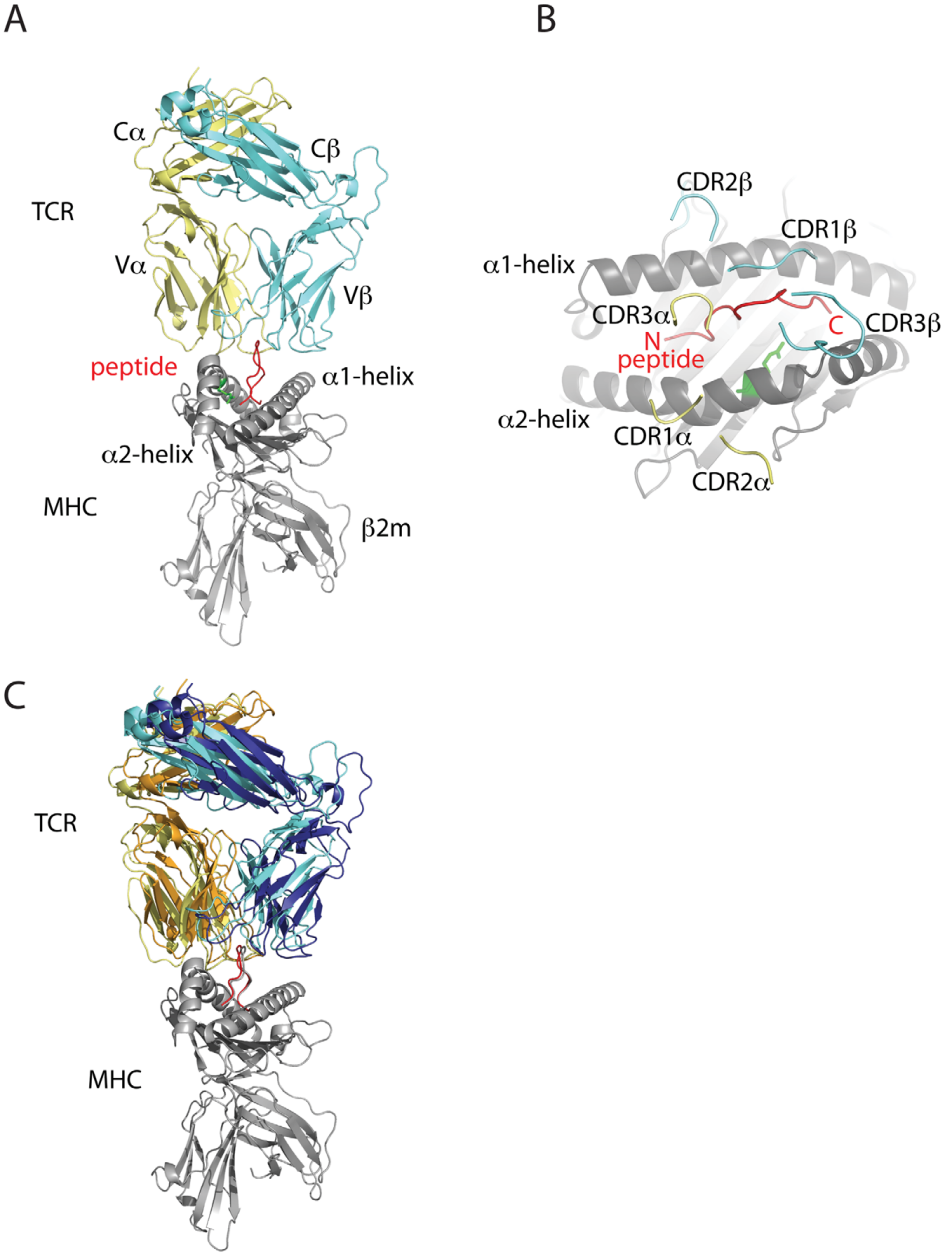
Overall cartoon of TCR-pMHC complex SB27-HLA-B*3508-LPEP (PDB ID 2AK4). (A) HLA-B*3508 shown in grey with polymorphic residue (R156) in green, LPEP peptide shown in red, SB27 TCR shown in yellow and cyan for α- and β- chains respectively; (B) Superposition of the two distinct TCR-pMHC complexes within the crystal structure, SB27 α- and β-chains of complex A coloured orange and dark blue respectively and complex B coloured as per (A); (C) aerial view of antigen binding cleft of HLA-B*3508-LPEP with colours as per (A) and the SB27 TCR removed with the exception of the CDR loops.

αβ T cell receptors (TCR) expressed on the surface of CD8+ T cells generally recognise specific antigenic or aberrant peptide fragments (usually 8–10 mers) that are bound to a cell surface MHC class I molecule. This interaction is central to adaptive T cell mediated immunity and induces signalling that results in T cell proliferation and differentiation into effector and memory T cells [1]. As such, the interaction between a TCR and cognate peptide-MHC (pMHC) is of great interest and soluble proteins have been studied extensively predominantly using X-ray crystallography combined with biophysical studies [2]. These studies have revealed some general trends. The Vα and Vβ domains of the TCR each comprise three complementarity determining regions (CDRs) which specify the antigen-binding site (Figure 1A). The CDRs from the Vα and Vβ chain are positioned over the antigen-binding cleft of the MHC which is flanked by the α1- and α2-helices (Figure 1A). Specifically the Vα and Vβ chains of the TCR sit atop the amino- (N-) and carboxy (C-) terminal residues of the peptide respectively (Figure 1B). Generally in this conserved binding orientation the hypervariable CDR3 loops contact the peptide, whereas the germline-encoded CDR1 and CDR2 loops contact the MHC helices [3]. It is likely that evolutionarily conserved interactions between certain amino acids in the CDR1 and CDR2 loops and the MHC backbone govern the conserved binding orientation of the TCR atop the MHC, allowing some flexibility in the binding angle and exact positioning of the TCR [4]–[8].

Intriguingly TCR-recognition of pMHC can be both degenerate and highly specific. For example, the TCR can recognise structurally different MHC-bound peptides yet also discriminate between peptides and MHCs that differ by a change as slight as a single polymorphism. This anomaly may be influenced by several factors including the extent of initial interactions made between the TCR-pMHC, modest changes at the TCR-pMHC interface that facilitate improved binding and stabilisation of the TCR-pMHC interaction [9] and the half-life of a productive interaction [10]. TCR-pMHC interactions typically have a slow association rate of binding (typically 10^3^–10^5^ M^−1^.s^−1^) [11], whereas the dissociation rate (0.1–100 s^−1^) is typically rapid and these kinetics are characteristic of a low affinity interaction [11]. There is substantial evidence that the *in vitro* and *in vivo* binding kinetics and efficiency of the TCR-pMHC interaction is correlated with the nature of the T cell response [12]. This suggests the TCR must engage the pMHC long enough to transmit a biological signal across the T cell membrane [13]–[16] and further studies have shown that successive TCR engagement leads to cumulative signalling and T cell activation [12]. Despite the existence of general rules of TCR-pMHC engagement, two pertinent questions remain unanswered; what guides MHC restriction, and how does TCR-pMHC binding lead to T cell activation?

While X-ray crystallography is a powerful technique for visualising molecular structures such as pMHC and TCR, the structure determined represents a space and time average of all molecules in the crystal lattice. Therefore information about the flexibility of the molecule is limited and can only be gained from the atomic temperature (B) factors of crystallographic models, however these values must be treated cautiously. As flexibility and dynamics of TCR-pMHC interactions may play an important role in governing their biological function, X-ray crystallography offers limited insight into these processes. However this information can be obtained computationally using the X-ray crystal structure combined with molecular dynamics (MD) simulations. The simulations offer the ability to calculate the time dependent behaviour of a molecular system over time, extending the information gained from crystallographic data and in this instance offering insights into the role of dynamics in a particular TCR-pMHC system.

Previous research has shown that an unusually long 13 mer peptide from the BZLF1 antigen of Epstein-Barr virus (amino acids 52–64, sequence LPEPLPQGQLTAY abbr LPEP) could bind HLA-B*3501 and HLA-B*3508 [17], but only HLA-B*3508-LPEP generates a robust and biased CTL response. Studies using T cells of a dominant clonetype (SB27) and the recombinant SB27 TCR indicated that SB27 could discriminate between HLA-B*3501-LPEP and HLA-B*3508-LPEP and yet they differ by only by a single polymorphism located on the α2-helix of the MHC (HLA-B*3508^R156^; HLA-B*3501^L156^). A structural comparison of the pMHCs revealed localised conformational changes accommodating the polymorphic residue, causing a 1 Å rigid body shift in the α2-helix from residues 145–158 originating from Val^152^. This shift caused the broadening of the HLA-B*3508-LPEP antigen binding cleft and was postulated to favour the interaction of the SB27 TCR [18]. Further, the SB27-B*3508-LPEP structure revealed that the polymorphic residue (Arg156) did not contact the TCR and hence could not directly influence T cell selection. The structure of the SB27-HLA-B*3508-LPEP also revealed an orthogonal docking mode of TCR-pMHC binding that unconventionally was dominated by peptide-mediated contacts with SB27 due to the length and bulged nature of the peptide. The limited MHC-mediated contacts that were made were described as the minimal residues required to produce a productive interaction and included residues Gln65 and Thr69 on the α1-helix and Gln155 on the α2-helix (referred to as the ‘restriction triad’) [19]. Further, comparison of the different complexes within the crystal asymmetric unit suggested a ‘rocking’ motion for the TCR in the two complex crystal forms (Figure 1C). While these studies provided valuable insights into the discriminatory ability of SB27, the dynamics of HLA-B*3508-LPEP and HLA-B*3501-LPEP and relevance to T cell selection have not been explored. To further comprehend the differential recognition of SB27 for HLA-B*3501-LPEP and HLA-B*3508-LPEP we performed MD simulations of both unliganded pMHCs and also HLA-B*3508-LPEP in complex with SB27. These studies reveal differences in the epitope dynamics between the unliganded pMHC structures that may govern the subsequent encounter with SB27. In addition we observe intriguing dynamics at the TCR-pMHC interface that may be indicative of the proposed mechanism of TCR scanning. Taken together, these results may provide significant insight into the molecular principles governing the role of epitope flexibility in SB27 recognition and set the precedent for similar studies of other TCR-pMHC systems.

## Results/Discussion

### MD reveals greater flexibility of the peptide compared to the MHC

**Figure 2:**
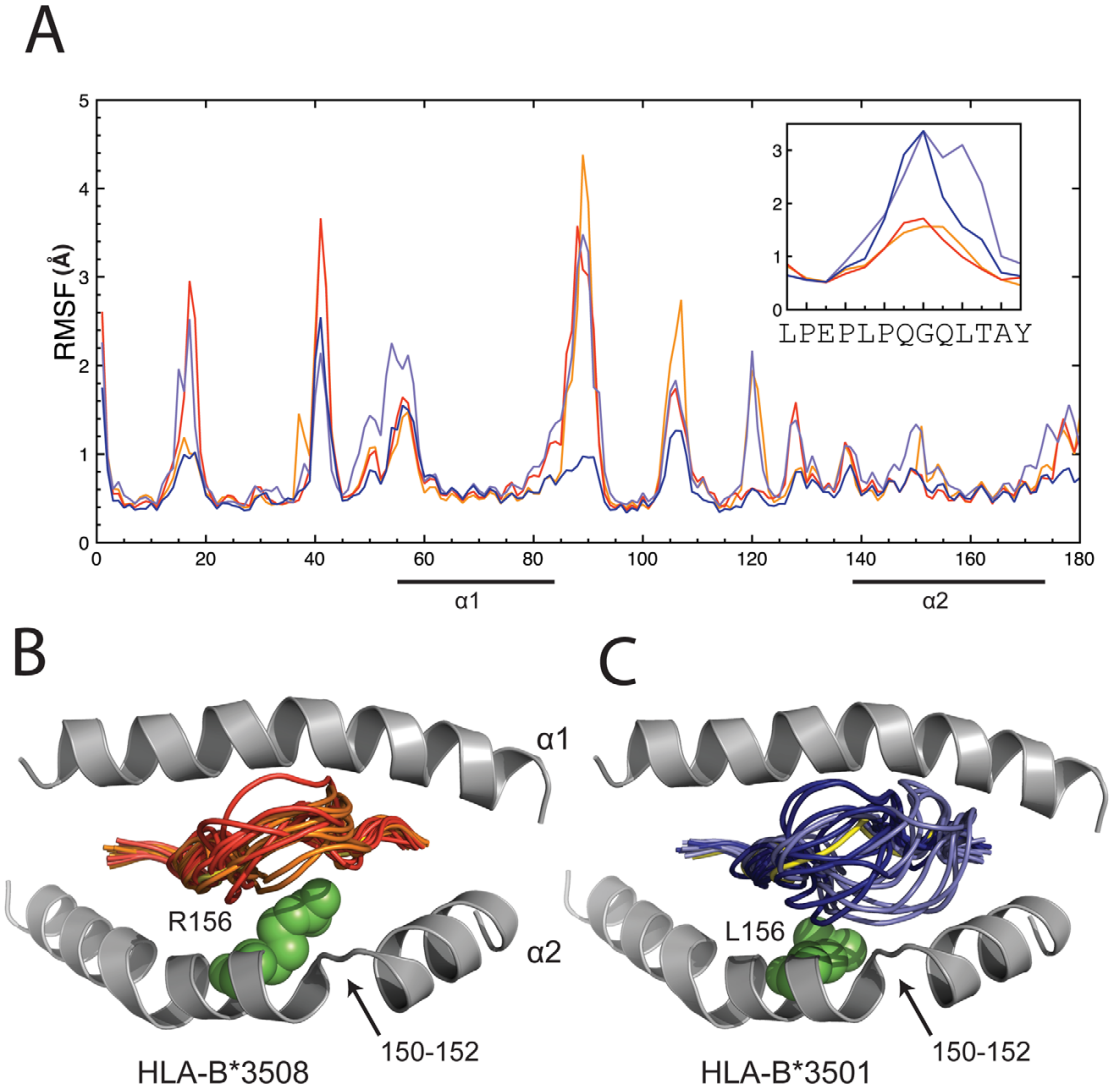
MHC and peptide conformational flexibility in MD simulations. (A) RMSFs as calculated from MD simulations for MHC and peptide residues (inset). Only positions 1 to 180 of the MHC molecules are displayed with the MHC helices underlined. HLA-B*3508 simulations 1 and 2 are in red and orange respectively and HLA-B*3501 simulations 1 and 2 are in dark and light blue respectively. The boxed inset highlights the RMSFs of the peptide at positions 1 to 13; (B) Cartoon representation of the crystal structure of HLA-B*3508-LPEP (PDB ID 1ZHL) with helices coloured grey, peptide in yellow and polymorphic residue in green. MD snapshots of the peptide taken every 10 ns and colour coded in red for simulation 1 and orange for simulation 2; (C) Cartoon representation of the crystal structure of HLA-B*3501-LPEP (PDB ID 1ZHK) with helices shown in grey, peptide in yellow and polymorphic residue in green. MD snapshots taken each 10 ns are colour coded dark blue for simulation 1 and light blue for simulation 2.

The conformation of the bound LPEP peptide is almost identical in the crystal structures of HLA-B*3501 and HLA-B*3508 (RMSD = 0.24 Å over 13 Cα atoms). We assessed the conformational flexibility of the unusually long LPEP peptide bound to MHC in both HLA-B*3501 and HLA-B*3508 using molecular dynamics (Figure 2 and Video S1). In both cases the amino acids at the termini of the peptide were immobile, consistent with their role in anchoring the peptide into the A- and F- pockets of the MHC respectively [20]–[22]. Whilst the termini of the peptides were consistently anchored, the peptide in both alleles was considerably more mobile than the MHC helices (Figure 2A). However, the simulations showed a marked difference in the conformational flexibility of the bound peptide depending on the MHC allele to which it was bound. When bound to HLA-B*3508 the peptide exhibited moderate flexibility (RMSF ∼1.5 Å; Figure 2A, B), which is also similar to the conformation seen when HLA-B*3508-LPEP is bound to the SB27 TCR. However when the peptide was bound to HLA-B*3501 the peptide mobility increased two-fold, particularly at the peak of the bulge from residues from 5 to 11 (Figure 2A, C; Video S1). The difference in flexibility of the HLA-B*3501 versus HLA-B*3508-bound peptide is moderately consistent with differences in the average temperature factors of the peptide in the respective crystal structures (23.1 Å^2^ and 15.8 Å^2^ respectively [18]). These observations are also consistent with the finding that HLA-B*3501-LPEP is 4°C less thermostable than HLA-B*3508-LPEP [18].

Given the high structural similarity between the two allelic MHC variants, we speculate that the difference in peptide flexibility when bound to the two alleles may be attributed to the polymorphic residue at position 156. In HLA-B*3508 the arginine at position 156 forms two water-mediated bonds with the LPEP peptide, including Glu3 O-ε1 and Thr11 O [18]. In HLA-B*3508 these bonds may tether the peptide in place, but in HLA-B*3501 the shorter leucine side chain at position 156 causes the loss of these bonds and the formation of a cavity [18]. This increase in space available may permit the LPEP peptide to adopt alternative conformations not seen in the crystal structure. Indeed, our simulations show that when bound to HLA-B*3501 the LPEP peptide predominantly fluxes between the conformation observed in the crystal structure (Figure 2C, yellow) and conformations that approach the hinge region of the α2-helix (residues 150–152; Figure 2C). Peptide flexibility can be experimentally tested using biophysical techniques, for example site directed fluorescence labelling [23], [24] and IR spectroscopy [25]–[27].

### Epitope flexibility in HLA-B*3501-LPEP frustrates or obstructs TCR recognition

Since the conformational variability corresponds to the central bulged region of the peptide and most of these residues (residues 4–9) are directly involved in TCR binding [19], the simulations results imply that peptide flexibility may influence T cell selection. Importantly this possibility could not be appreciated from the crystal structure alone (Figure 2B). Peptide flexibility may frustrate the ability of SB27 to form critical contacts with LPEP residues 4–9. Alternatively or additionally, peptide flux towards the hinge region of the α2-helix (residues 150–152) may sterically obstruct the CDR3β loop of SB27 from contacting the MHC. (Figure 1B). In the SB27-LPEP-HLA*B*3508 complex, the CDR3β loop forms Van der Waals interactions with residues near the hinge-region of the α2-helix, including Ala150, Arg151, Glu154, Gln155, and a salt-bridge with Arg151 [19]. Of note, three of these four CDR3β residues (151, 154 and 155) are frequently used by TCRs to contact the MHC [5]. Further, due to the bulged nature of the LPEP peptide these MHC residues account for a substantial four out of six that are contacted by SB27 [19]. Therefore it is likely that the obstruction of these interactions would have a significant effect on TCR binding. Accordingly the affinity of SB27 for HLA-B*3501-LPEP is >3-fold weaker than for HLA-B*3508-LPEP and dissociates approximately 2-fold faster [18], indicating critical interactions are indeed lost.

Whilst TCRs are able to recognise inherently flexible epitopes [28], [29] it is possible that the flexibility of the LPEP bound to the HLA-B*3501 may play an under-appreciated role in SB27 binding. Based on the inability of SB27 to adapt to a more highly flexible but otherwise identical epitope we predict the CDR loops of SB27, whilst sufficient to adapt to a wide range of epitope structures, are “conformationally frustrated” or sterically obstructed by a highly flexible moving peptide target. Our data shows this discrimination may be due to residues at the periphery of the SB27-HLA-B*3508-LPEP interface. It is possible that conformational plasticity of SB27 is fine-tuned to allow discrimination between such conformationally diverse but otherwise identical epitopes, contributing to the exquisite specificity of SB27 in LPEP antigen recognition. Indeed, it has been suggested that antibodies, which have limited sequence diversity, use conformational diversity to achieve functional promiscuity [30].

### MD reveals insight into possible scanning motion of TCR on pMHC

**Figure 3:**
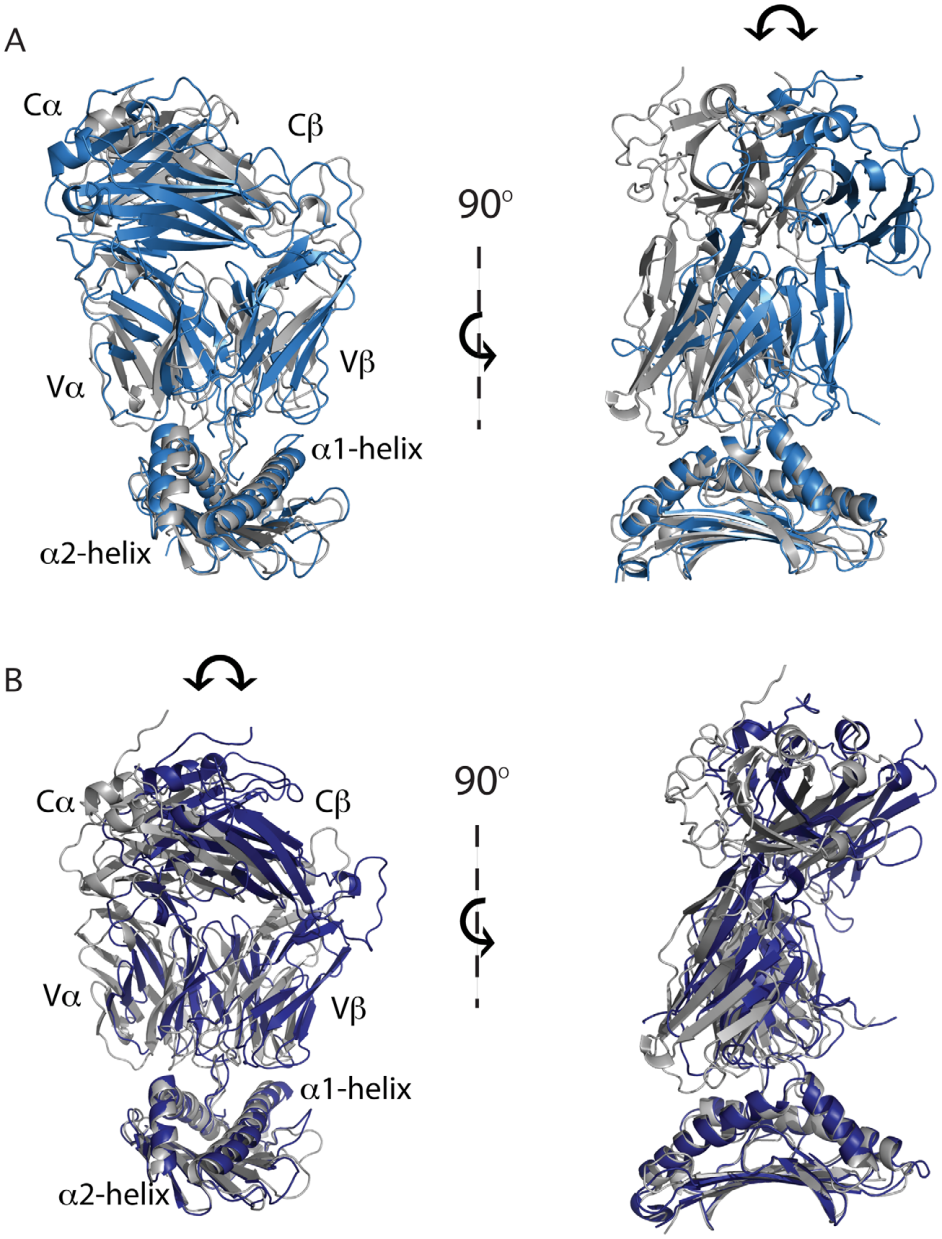
TCR-pMHC scanning. The crystal structure of (A) complex A and (B) complex B is in grey for reference and the maximum movement of the TCR during the respective simulations is superposed in light blue or dark blue. Orthogonal orientations are shown in the right hand panels. The arrow highlights the direction of TCR movement during the respective simulations.

Interestingly, and as previously published, four SB27-HLA-B*3508-LPEP complexes were present in the asymmetric unit [29]. While the individual components were almost identical, two subtly different TCR-pMHC conformations were revealed (Figure 1C), as also observed in the JM22-HLA-A*0201-flu crystal forms [31]. The difference was attributed to a 12° rotation in the angle of approach by the TCR consistent with the formation of new bonds at the interface, and the destruction of others. As crystal contacts were deemed an unlikely cause of this phenomenon, it was postulated that the tilted orientation of the TCR on the pMHC may offer a glimpse into the scanning mechanism of the TCR [19]. Using both TCR-pMHC complex conformations as starting points for MD simulations we show that both complex conformations exhibit a rocking trajectory that extends beyond the two positions observed in the crystal structure (Figure 3). However, the simulations revealed that the rocking trajectory of the TCR in the two complexes was inconsistent. In complex A the TCR motion is predominantly parallel to the antigen-binding cleft (Figure 3A and Video S2) whereas in complex B the TCR movement is predominantly orthogonal to the antigen-binding cleft (Figure 3B). There is evidence that the crystalline environment can select muliple distinct biologically relevant conformations from an ensemble observed in solution [32]–[37], and that cryogenic temperatures of data collection can further bias the observed conformers [38]. Our simulation results are consistent with this view. This pronounced rocking motion may be due to the bulged and protruding nature of the LPEP peptide which has been shown to limit MHC-TCR interactions [19]. Nonetheless, this phenomenom may also provide a valuable insight into the physiological importance and stochastic nature of SB27 TCR scanning. TCRs may scan the pMHC surface to rapidly differentiate between the numerous combinations encountered [39]. It may also provide a mechanism for the TCR to disengage from the pMHC.

### The TCR pivots atop residues 6–9 of the LPEP peptide

Given the protruding nature of the LPEP peptide, the SB27 TCR contacted minimal HLA-B*3508 residues as observed in the crystal structure [19]. These minimal residues, termed the ‘restriction triad’ (Gln65, Thr69, Gln155) [19] are repeatedly contacted by TCRs in complex with peptide-MHC class I. Our MD data supports the observation that contacts with the restriction triad are made, but indicates that these contacts fluctuate and may only be transient (Table 1 and Video S3). The transient nature of these interactions is supported by mutational studies indicating these residues are non-essential to SB27 CTL killing [40] and SB27 binding affinity [40]. Furthermore, the crystal structure of SB27 in complex with LPEP-HLA-B*3508 harbouring the triple alanine mutation of the restriction triad shows that SB27 forms compensatory interactions and exhibits an altered docking mode indicative of an adaptable TCR [40]. MD reveals that the SB27 TCR makes relatively long-lived contacts with Pro6, Gln7, Gly8 and Gln9 of the bulged LPEP peptide (Video S3), suggesting the bulged region of the peptide is crucial to the interaction and also a pivot point of the TCR. The importance of Pro6, Gln7 and Gly8 is reflected by a glycine substitution analysis whereby individual substitution to glycine results in significantly reduced CTL recognition [18]. Also, whilst the triple alanine substitution of LPEP-HLA-B*3508 causes compensatory MHC interactions that shifts SB27 docking, the contacts with Pro6, Gln7, Gly8 and Gln9 of the LPEP peptide predominate and constitute 32 of the 38 interactions made with the TCR [40]. Therefore taken together the biological data, structural data and molecular dynamics cumulatively reveal that the SB27 TCR is indifferent to alterations in the restriction triad residues of the MHC but highly sensitive to changes in Pro6, Gln7 and Gly8 of the peptide given the long-lived nature of these interactions. The peptide-centric nature of the SB27 interaction may be due at least in part to the unusually long and prominent LPEP-peptide.

**Table 1:** Hydrogen bonds between the pMHC and the TCR observed over the course of the simulations.

MHC	TCR	In crystal structure?	Persistence Complex A (% of simulation)	Persistence Complex B (% of simulation)
α**1-helix**				
Arg62^Nη1/2^	CDR2β Asp56^Oδ1/2^	No	54	-
Gln65^Nε2^	CDR2β Ser51^Oγ^	No	-	10
Gln65^Nε2^	CDR2β Glu52^Oε1/2^	No	-	27
Gln65^Oε1^	CDR3α Asn97^Nδ2^	Yes	23	5
Gln65^Oε1^	CDR2β Glu52^N^	No	7	-
Lys68^Nζ^	CDR2β Glu52^Oε1/2^	No	48	39
**α2-helix**				
Arg151^Nη1/2^	CDR2α Asp53^Oδ1/2^	No	46	-
Arg151^Nη1/2^	CDR2α Glu57^Oε1/2^	No	-	36
Glu154^Oε1^	CDR2α Arg48^Nη2^	No	-	7
Glu154^Oε1^	CDR2α Asn50^Nδ2^	No	15	9
Glu154^Oε2^	CDR2α Asn50^Nδ2^	Yes	14	6
Gln155^Nε2^	CDR3α Phe95^O^	Yes	3	1
Gln155^O^	CDR3α Tyr96^Oη^	No	-	15
Arg157^Nη1/2^	CDR2α Glu54^Oε1/2^	No	-	27
**Peptide**				
Leu5^O^	CDR3α Asn97^N^	Yes	60	52
Pro6^O^	CDR1β Asn30^Nδ2^	Yes	42	18
Gln7^Nε2^	CDR3α Tyr96^O^	No	63	46
Gln7^Nε2^	CDR3α Ser93^Oγ^	Yes	17	25
Gln7^O^	CDR1β Asn30^N^	Yes	4	-
Gln7^O^	CDR1β Ser31^N^	Yes	15	61
Gln7^Oε1^	CDR1α Tyr31^Oη^	Yes	66	39
Gln7^O1^	CDR3α Ser93^Oγ^	Yes	21	25
Gln7^Oε1^	CDR1β Tyr33^Oη^	Yes	-	5
Gly8^O^	CDR1β Asn30^N^	Yes	77	35
Gly8^O^	CDR1β Asn30^Nδ2^	Yes	13	18
Gln9^N^	CDR1β His29^Nδ1^	Yes	1	1
Gln9^Nε2^	CDR1β Asn28^O^	No	7	15
Gln9^Nε2^	CDR1β Asn28^Oδ1^	Yes	-	-
Gln9^Nε2^	CDR1β His29^Nδ1^	No	35	16
Gln9^Oε1^	CDR3β Tyr103^Oη^	No	-	5

(-) indicates no interaction observed in simulation.

### TCR binding restricts flexibility of peptide pivot residues but not MHC

**Figure 4:**
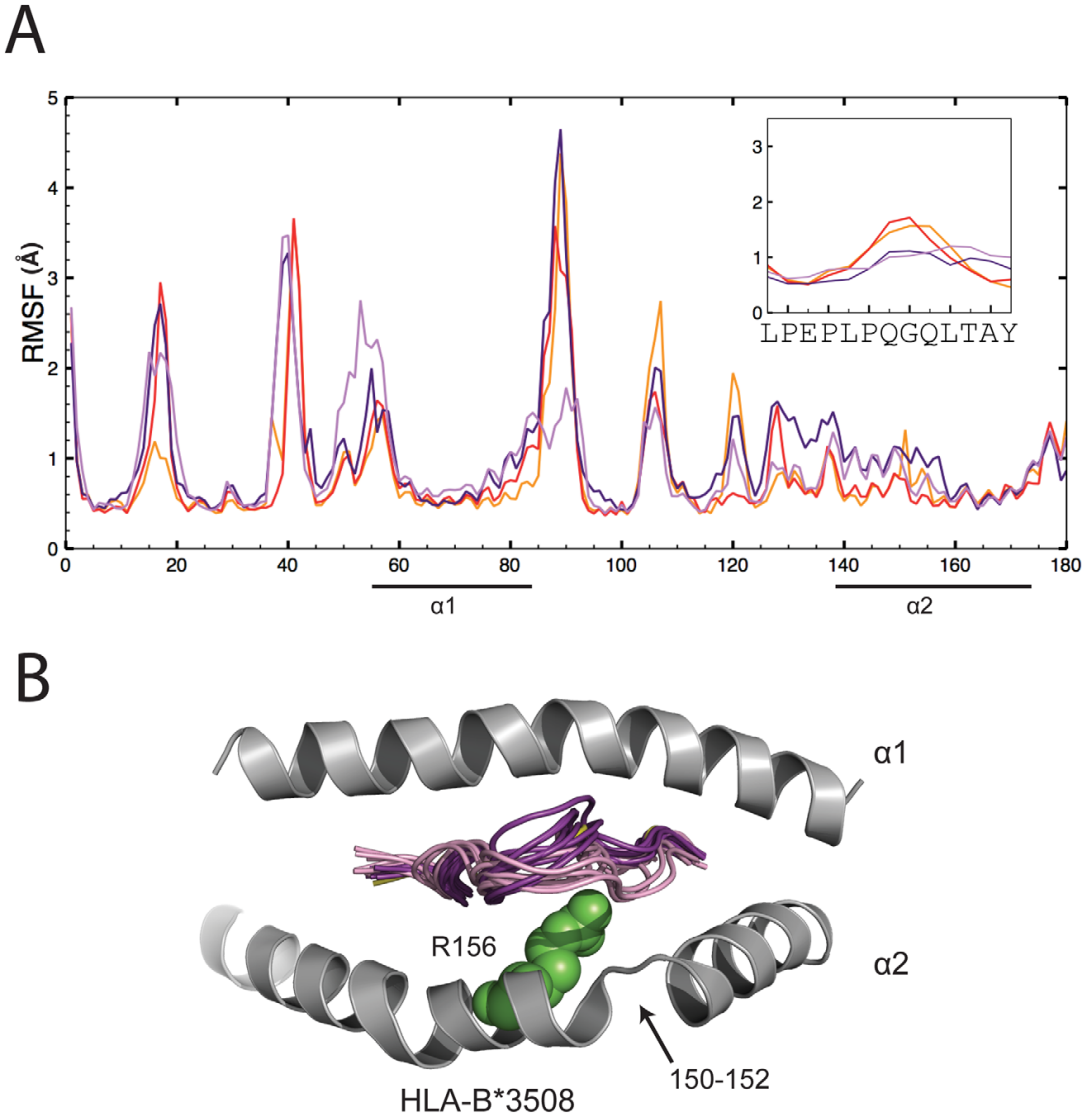
The flexibility of the LPEP peptide within HLA-B*3508 while complexed to the SB27 TCR. (A) RMSFs as calculated from MD simulations for MHC and peptide residues. Only positions 1 to 180 of the MHC molecules are displayed with the MHC helices underlined. HLA-B*3508-LPEP simulations 1 and 2 are in red and orange respectively and HLA-B*3508-LPEP SB27 TCR simulations 1 and 2 are in dark purple and pink respectively. The boxed insert highlights the RMSFs of the peptide at positions 1 to 13; (B) Cartoon representation of the crystal structure of HLA-B*3508-LPEP SB27 TCR complex (PDB ID 2AK4) with helices coloured grey, peptide in yellow and polymorphic residue in green. For clarity the SB27 TCR has been removed. MD snapshots of the peptide taken every 10 ns are colour coded as per A.

As an intuitive control and to validate our molecular dynamics simulations we compared the flexibility of the LPEP peptide when bound to HLA-B*3508 alone (Figure 2A, B) and in complex with SB27 (Figure 4A, B). In the absence of SB27 we observe flexibility of peptide residues 5–11 (RMSF ∼1.5 Å; Figure 2A, B), however upon complexation the flexibility of the pivot residues (6–9) is reduced (RMSF ∼1.0 Å; Figure 4A, B) due to their long-lived hydrogen bonds with TCR. In contrast, TCR binding does not significantly alter the flexibility of MHC contact residues (Figure 4), again highlighting the significance of the prominantly bulged peptide pivot for the TCR interaction.

### A dynamic pMHC-TCR footprint

**Figure 5:**
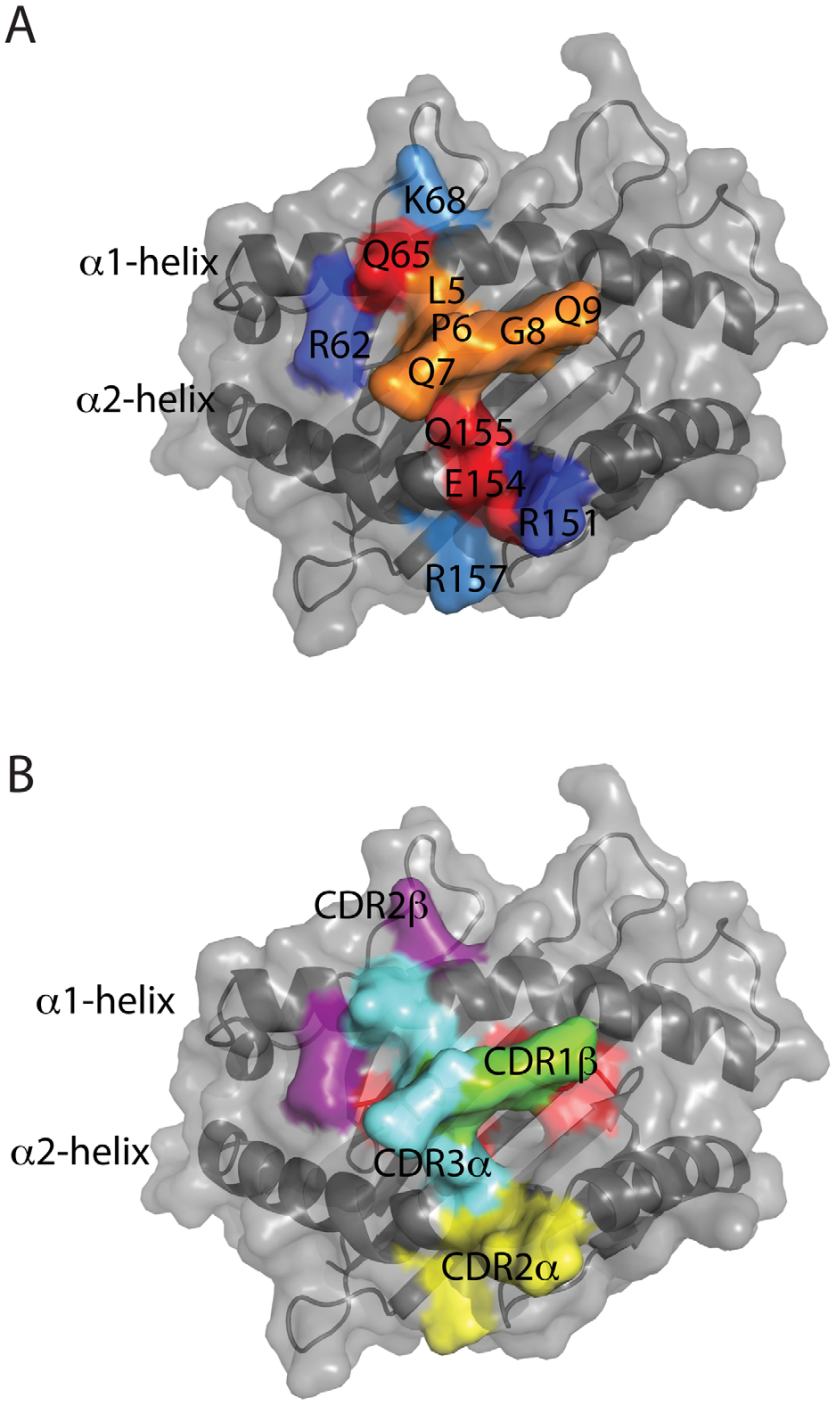
The SB27 TCR footprint on HLA-B*3508-LPEP following MD simulation. (A) A comparison of contacts observed in both the crystal structure and dynamics. MHC or peptide contacts observed in both the crystal structure and dynamics coloured red and orange respectively. Residues coloured in light or dark blue represent novel short-lived (<50%) or long-lived (>50%) interactions respectively that were observed only during the simulation. The footprint represents both complex A and complex B as all contacts were conserved between the two simulations with the exception of Arg157 which was a short-lived contact only observed in the simulation of complex A and Arg151 and Arg162 which were long-lived contacts only observed in the simulation of complex A; (B) The HLA-B*3508 and LPEP residues contacted by the TCR are coloured according to the CDR loop from which they are derived. The uncontacted regions of LPEP are in red, residues contacted by CDR2α in yellow, CDR3α in cyan, CDR1β in green and CDR2β in purple.

A detailed inspection of the pMHC-TCR interface during the simulation of SB27-HLA-B*3508-LPEP reveals both short- and long-lived interactions that occur during the scanning motion described above (Table 1, Figure 5 and Video S3). We were thus able to construct a TCR-pMHC footprint that encompasses the known footprint from crystallographic studies but also encompasses additional interactions that may play a role during or even dictate the lifetime of the interaction. While the simulations revealed the TCR in each complex adopts a dissimilar rocking trajectory, both maintained a conserved footprint of contact residues on the pMHC (Table 1, Figure 5 and Video S3). This indicates that the TCR contacts the same residues on the pMHC, but does so randomly rather than adopting a defined pathway. However in addition to the interactions seen in the crystal structure that also persist in the simulation, several new and persistent hydrogen-bonds were identified that are not present in the crystal structure. Therefore, over the course of the simulation the SB27 samples additional, remote regions of the α1- and α2-helix of HLA-B*3508-LPEP (Figure 5A, blue regions). In particular, in the crystal structure the side chain of Arg62 (α1-helix) does not interact with either LPEP or SB27, but early in the simulation undergoes a significant conformational change to form a salt bridge with Asp56 (SB27 CDR2α; Table 1 and Video S3). It is interesting to note that these additional MHC contacts are made by residues in the germline-encoded CDR2α and CDR2β loops (Table 1, Figure 5B, and Video S3). MD simulations on other TCR-pMHC systems may reveal that these loops play an even greater role in MHC-restriction than previously anticipated. In this instance however, the interactions may represent additional hot-spots that dictate the SB27-LPEP-B*3508 dynamics, half-life and therefore T cell signalling. The role of these residues could be tested experimentally using site directed mutagenesis coupled with surface plasmon resonance.

### Dynamic versus static views of TCR-pMHC interactions

The effect of crystal packing on protein structure has been studied widely by comparing identical molecules crystallized in different lattices [32]–[36]. Indeed in the case of T4 lysozyme, radically different domain conformations of four molecules in the crystal lattice have been reported [32], suggesting that crystal contacts can select distinct conformations from an ensemble and may provide hints to conformational dynamics. Similarly, two different conformations were observed in the crystal structure of the TCR-pMHC complexes that formed the basis of our study. Our MD results extend the range of these motions, suggesting that in this case the crystal lattice may have selected compatible conformations from a diverse ensemble. Furthermore, cryo-cooling can result in smaller, over-packed models [38], with significantly reduced or even eliminated motions important for protein function. This highlights the need for complementary methods, such as MD that provide important insights into the role of conformational heterogeneity in TCR-pMHC interactions and signalling outcome. This will require characterisation of all molecular species and assemblies at the synapse in the context of APC T-cell interaction. However, the structural characterisation of relevant molecular assembles (e.g., including co-receptors CD8/CD4, the CD3 complexes, and other co-stimulatory factors) is likely to provide only a static snapshot of a pre-signalling situation, and our data suggests that a complete understanding of the signalling events will require a full characterisation of the dynamics of the process.

In summary, our results reveal a striking contrast between the dynamic behaviours of the LPEP peptide captured by HLA-B*3508 compared to HLA-B*3501. The LPEP peptide was found to be more flexible when bound to HLA-B*3501 versus HLA-B*3508 and this was attributed to a single polymorphism within the peptide-binding cleft of the MHC. Notably the inherently flexible nature of the LPEP peptide in both HLA-B*3501 and HLA-B*3508 could not be appreciated from the crystal structures alone but the marked difference in LPEP flexibility in the two allotypes may influence subsequent interaction with the SB27 TCR. On the basis of these results we predict that the flexibility of the SB27 TCR, although sufficient to adapt to a wide range of HLA-B*3508-LPEP conformations, is unable to engage productively with a highly flexible peptide, thus causing “conformational frustration” and a non-productive LPEP-HLA-B*3501 interaction. This may indicate that peptide dynamics plays an influential role in SB27 T cell selection and activation. Interestingly, unexpected conformational plasticity in the TCR, which may play a role in binding different pMHC has been recently reported [41]. Further, the pivoting motion of SB27 atop HLA-B*3508-LPEP provides insights into possible scanning mechanisms that SB27 may use to distinguish between combinations of pMHC landscapes. To our knowledge this is the first description of TCR scanning of a cognate pMHC surface using a molecular dynamics approach. Taken together, these simulations provide several insights into TCR-pMHC interactions that are not available using crystallography. This study sets the precedent for molecular dynamics simulations of other TCR-pMHC systems. Given the unconventional length of the LPEP peptide, it will be interesting to note whether our observations are particular for the SB27-LPEP-HLA-B*3508/B*3501 system or are reflective of other, perhaps more conventional TCR-pMHC systems. Four related studies, incorporating MD and biophysical techniques have shown that peptide flexibility, not apparent in the crystalline environment is also observed with shorter more conventional length peptides [23]–[27], [42]. This may indicate that peptide flexibility may also be a feature of shorter peptides, and may play an important role in T-cell recognition.

## Methods

The simulations were based on the published crystal structures of HLA-B*3501 and HLA-B*3508 each with bound LPEP peptide (LPEPLPQGQLTAY) (PDB IDs 1ZHK and 1ZHL respectively) [18], and on the crystal structure of the complex of HLA-B*3508 with the TCR SB27 (PDB ID 2AK4) [19].

The HLA-B*3508-LPEP-TCR complex contains 4 complexes in the asymmetric unit, with 2 unique conformations designated Complex A and Complex B, where Complex A comprises chains K-P and Complex B chains A-E. Missing residues were added to the structure using Modeller, version 9v6 [43]. The two distinct crystal structure conformations of the pMHC-TCR complex were both used as distinct starting points for the molecular dynamics study of the complex. For the simulations of pMHC-LPEP complexes the single molecule in the asymmetric unit of the crystal was used for HLA-B*3501 (PDB ID 1ZHK) and for HLA-B*3508 (PDB ID 1ZHL).

MD simulations were performed using the NAMD 2.7 MD software package [44] and the CHARMM22 protein force field [45]. The structures were solvated with waters (TIP3P model) and 0.2 mM NaCl, then subjected to energy minimization. For all simulations a timestep of 2 fs was used as well as periodic boundary conditions and PME for electrostatics. The systems were heated to 300 K with the protein harmonically constrained for 0.4 ns followed by constraining Cα atoms only for 0.8 ns. The systems were equilibrated for another 1 ns without constraints before the production runs used for analysis. Parameters for all simulations were: temperature 300 K, switching distance 10 Å, switching cutoff 12 Å, pairlist distance 13.5 Å, Langevin damping coefficient 5 ps^−1^, Langevin pressure control with a target pressure of 1.01325 bar.

The systems subjected to MD simulations are as follows:

HLA-B*3501-LPEP: Cubic box with initial dimensions 79.2×95.7×74.0 Å and PME grid dimensions 80×96×74 Å. The system contained 51887 atoms, consisting of 15211 waters, 57 ions (34 Na^+^ and 23 Cl^−^) and 6197 protein atoms, resulting in a chargeless system. The system was used as the starting point for two independent simulations of 100 ns each.

HLA-B*3508-LPEP: Cubic box with initial dimensions 79.2×95.6×74.3 and PME grid dimensions 80×96×76. The system contained 52016 atoms, consisting of 15252 waters, 58 ions (34 Na^+^ and 23 Cl^−^) and 6202 protein atoms, resulting in a chargeless system. The system was used as the starting point for two independent simulations of 100 ns each.

SB27-HLA-B*3508-LPEP (*Complex A and Complex B*): Cubic box with initial dimensions of 96 Å×86 Å×162 Å and PME grid dimensions 96×90×162. The whole system contained 137926 atoms, consisting of 41502 water molecules, 156 ions (90 Na^+^ and 66 Cl^−^), 13264 protein atoms in 844 protein residues resulting in a chargeless system (Complex A). Cubic box with initial dimensions of 96 Å×86 Å×162 Å and PME grid dimensions 100×90×162. The whole system contained 137872 atoms, consisting of 41484 water molecules, 156 ions (90 Na^+^ and 66 Cl^−^), 13264 protein atoms in 844 protein residues (Complex B). Simulations for both complexes A and B were performed for 100 ns each.

All analyses and visualisations were performed using VMD [46], PyMOL [47], and custom scripts. Hydrogen bonds were considered formed for a donor/acceptor cut-off distance of 3.5 Å and an angle with the central hydrogen of 180±30°. RMSF profiles presented in figure 2A and 4A employed Cα positions of MHC residues 1 to 180 and residues 1 to 13 of the peptide, with respect to the average structure.

## Supporting Information

Video S1 Conformational flexibility of peptide in HLA-B*3501-LPEP and HLA-B*3508-LPEP during MD simulations. The polymorphic (R/L156) residue is shown in green. Static snapshots of the respective simulations are shown in the insets as per Figure 2B, C.
(MP4)

Video S2 Orthogonal views of MD simulation of HLA-B*3508-LPEP SB27 TCR complex B. The MHC, LPEP epitope and TCR are shown in grey, green and yellow/cyan respectively.
(MP4)

Video S3 Detailed view of contacts made at the interface during the MD simulation of HLA-B*3508-LPEP SB27 TCR complex B. On the left, cartoon showing hydrogen bonds and salt bridges (black dotted lines) at the interface between MHC (grey), peptide (green) and TCR (cyan and yellow); On the right, MHC surface showing the dynamic TCR footprint. The MHC residues being contacted by the TCR are coloured red. The inset shows the corresponding footprint calculated from the crystal structure.
(MP4)
